# 

**Published:** 1893-09

**Authors:** 


CONTENTS—SEPTEMBER, 1893.—VOL. IX., NO. 3.

ORIGINAL COMMUNICATIONS—
Electricity in Medicine. Prof. Seth M. Morris, M. D., A. B., Galveston . . . . . 99
Pelvic Abscess with Gangrene of the Vermiform Process. A. H. Schenck, M. D., Kenney . . . . . 113
Chloroform in Labor. J. Marion Dunagan, M. D., Barry . . . . . 119

ABSTRACTS OF CURRENT MEDICAL LITERATURE—
Department of Practice of Medicine. Edited by Prof. A. J. Smith, Galveston . . . . . 121-129
Department of Obstetrics and Gynecology. Edited by Prof. Wm. Keiller, M. D., Galveston . . . . . 129-131

SOCIETY NOTES—
Johnson County Medical Society . . . . . 132
Southeast Texas Medical Society . . . . . 133
Austin County Medical Society . . . . . 134

EDITORIAL—
Amick . . . . . 135
The "Sorry-Bird" of Hot Springs . . . . . 153

MEDICAL NEWS AND MISCELLANY—
Numerous paragraphs of general interest to the profession . . . . . 139

PUBLISHERS' NOTES . . . . . 146

